# Comparison of cardiac output, IVC diameters and lactate levels in prediction of mortality in patients in emergency department; An observational study

**DOI:** 10.12669/pjms.36.4.2032

**Published:** 2020

**Authors:** Kavous Shahsavarinia, Ali Taqizadieh, Payman Moharramzadeh, Ramin Amirchoupani, Ata Mahmoodpoor

**Affiliations:** 1 Kavous Shahsavarinia, Road Traffic Injury Research Center, Tabriz University of Medical Sciences, Tabriz, East Azerbayjan, Iran; 2 Ali Taqizadie, Lung Disease and Tuberculosis Research Center, Tabriz University of Medical Sciences, Tabriz, East Azerbayjan, Iran; 3 Payman Moharramzadeh, Emergency Medicine Research Team, Tabriz University of Medical Sciences, Tabriz, East Azerbayjan, Iran; 4 Ramin Amirchoupani, Department of Emergency Medicine, Faculty of Medicine, Tabriz University of Medical Sciences, Tabriz, East Azerbayjan, Iran; 5 Ata Mahmoodpoor, Department of Anesthesiology, Faculty of Medicine, Tabriz University of Medical Sciences, Tabriz, East Azerbayjan, Iran

**Keywords:** Cardiac output, Inferior vena cava, Lactate, Mortality

## Abstract

**Objective:**

Fluid overload is an independent marker for mortality in critically ill patients. Assessment of fluid status and fluid responsiveness is crucial for the management of these patients. In this study, we compared the lactate level, inferior vena cava (IVC) diameter and non-invasive cardiac output (CO) monitoring in prediction of mortality in emergency department.

**Methods:**

This was a cross sectional observational study which comprised of 68 patients and was performed in ED of Tabriz University of Medical Sciences, Iran, from Sept 2016 until Sept 2017. IVC diameter was measured before the P-wave on ECG to avoid interference with a-wave and v-wave on the venous pressure curve, and during maximal inspiration and expiration to avoid Valsalva-like maneuvers. An arterial lactate sample was taken from all patients before performing the initial resuscitation. All patients underwent non-invasive CO monitoring by CO_2_ rebreathing technique. Mortality was noted on day 28.

**Results:**

Deceased patients had a significantly low level of IVC diameters, less CO values and more lactate levels. However, based on ROC curve analysis, the prediction accuracy and validity of both CO values obtained by rebreathing CO_2_ and IVC diameter was poor and the highest accuracy was obtained by lactate level assessment.

**Conclusion:**

Initial lactate value is a reliable parameter for prediction of mortality in non-traumatic critically ill patients. IVC diameter changes during spontaneous ventilation and non-invasive CO monitoring does not possess acceptable accuracy for prediction of mortality in these patients.

## INTRODUCTION

As fluid overload is an independent risk factor for mortality, prediction of fluid responsiveness is an important way for reducing mortality in critically ill patients.^1^ Many markers have been introduced for prediction of fluid responsiveness and mortality in these patients.^2^ Elevated lactate has been found to be associated with a higher mortality rate in a vast patient population.^3^ A study showed that a serum lactate level of more than 2.6 mmol/lit predicted 30-day mortality in critically ill patients admitted to emergency department (ED).^4^ Dunham et al. showed that noninvasive cardiac output monitoring provides a useful clinical and objective method for cardiac output monitoring in critically ill patients admitted to ED.^5^ Hou et al. showed that in pre-shock patients admitted to ED, a fluid responsiveness protocol managed with non-invasive cardiac output monitoring, facilitated the delivery of more fluid administration.^6^ In previous trials, the inferior vena cava (IVC) diameter investigated by ultrasound was shown to predict intravascular volume in critically ill patients.^7^,^8^ Benefits of using ultrasound for IVC measurement include its non-invasiveness, rapidity and ease of use. However, its availability, body habitus, obstructing bowel gas, subcutaneous emphysema, and the variability of the results between operators are all known downfalls.^9^ There are many studies about the predictive value of IVC diameter variation in critically ill patients regarding fluid responsiveness with controversial results.^10^-^14^ Based on the mentioned studies, we wanted to compare the cardiac output values, IVC diameters and lactate levels as predictors of mortality in critically ill non-traumatic patients in ED.

## METHODS

This was a cross sectional observational study which comprised of 68 patients and was performed in ED of the largest university-affiliated hospital in North West of Iran. All critically ill non-traumatic patients who were admitted to ED during Sept. 2016 until Sept. 2017 were enrolled in this study. Inclusion criteria were requirement of fluid resuscitation and patients with spontaneous ventilation. Exclusion criteria were ages less than 18 years old, pregnancy, body mass index more than 30, preexisting severe valvular heart diseases or intracardiac shunts, cardiac arrhythmia and ascites. The ethics committee of Tabriz University of Medical Sciences approved (Ref.No: 5/5/8324, dated May 7, 2016) the protocol of the study and the written informed consent was taken from all patients or their next of kin. Sample size calculation was performed by Medcalc software. Considering type I error of 0.05, power of 80%, acceptable area under ROC curve of 0.75, null hypothesis value of 0.5 and positive to negative ratio of 0.25, sample size was estimated to be 64 patients which was increased to 68 patients in this study.

### Measurement of IVC diameter

The transducer was placed in the subxiphoidal region and long and short axis views of the IVC were obtained just below the diaphragm in the hepatic segment. An M-mode echocardiogram with simultaneous electrocardiographic monitoring was recorded. The IVC diameter was measured before P-wave on the ECG to avoid interference with a-wave and v-wave on the venous pressure curve. IVC diameter was measured during maximal inspiration and expiration, avoiding Valsalva-like maneuvers.^10^

### Measurement of serum lactate

An arterial lactate sample was taken from all patients after emergency medicine department admission and before performing initial resuscitation.

### Measurement of cardiac output

The CO_2_/volume sensor was connected between the rebreathing mask and the O_2_ circuit. The pulse oximeter was placed on the finger. The cardiac output measurements were performed every 3 minutes. Differences in CO_2_ elimination and end-tidal CO_2_ between the normal and rebreathing state were used to compute cardiac output, by a modification of the Fick equation.

All demographic characteristics, comorbidities, reasons for admission were noted for all patients. Lactate levels before and after resuscitation, cardiac output measurements and IVC diameters were noted for all patients. Mortality at 29 days was noted for all patients.

### Statistical analysis

All data were entered in statistical software package, SPSS 21 and were analyzed. Data were expressed as Mean± SD. T-test was used for comparison of quantitative variables and chai square test was used for qualitative variables. We used multi-logistic regression analysis for detection of correlation of each variable and with comparison of these correlations the relation of each variable with mortality was detected.

## RESULTS

**Fig.1:**
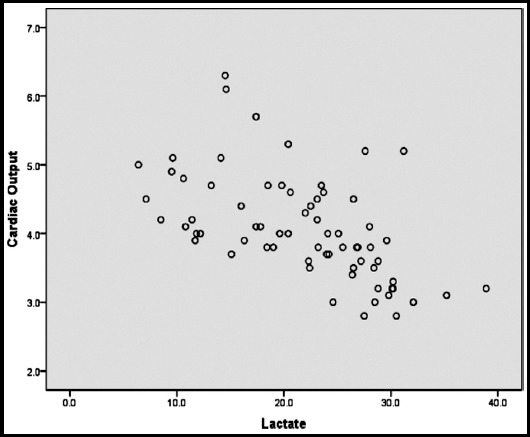
Correlation between cardiac output and lactate levels.

**Fig.2:**
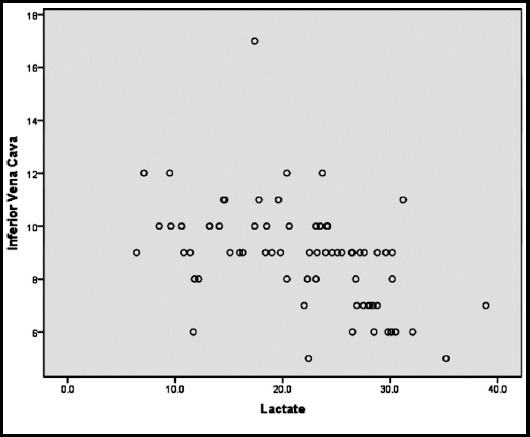
Correlation between inferior vena cava diameter and lactate levels.

Sixty-eight patients were enrolled in this observational study, 36 being male and 32 being female. The mean age of patients was 66.44±13.6 years old. Fifty-one patients survived and 17 were expired. We divided initial lactate level into two groups: lactate level more than 19.8 or less/equal than 19.8. Our results showed that 26 patients had lactate levels less than 19.8 and the other had a level more than that. Mean value for cardiac output measurement was 4.07 lit/min (2.8- 6.3). Mean value for IVC diameter was 8.5 mm (5-17mm). Pearson correlation coefficient between initial lactate and IVC diameter was -0.46. As the value is near to 0.5 we can conclude that there is a negative intermediate relation between the two variables. As the IVC diameter decreases due to depletion of intravascular volume, the level of lactate increases. The Pearson correlation coefficient between lactate level and cardiac output was -0.56 (Fig.1). Fig.2 shows the correlation between IVC diameter and lactate level. Correlation coefficient for IVC diameter and cardiac output values was 0.71 which showed a strong positive relationship between the two variables. Our results showed that expired patients had a significantly low level of IVC diameter, less cardiac output values and more lactate levels. ROC curve analysis showed the highest area under curve (91%) for lactate regarding prediction of mortality. The AUC for cardiac output and IVC diameter was 10.7 and 23.1, respectively. So, based on our results, the prediction accuracy and validity of both cardiac output value obtained by rebreathing CO_2_ and IVC diameter is poor.

## DISCUSSION

Our study results showed that initial lactate value in critically ill non-traumatic patients has more accuracy compared to cardiac output measurements obtained non-invasively by CO_2_ rebreathing and IVC diameter by ultrasonographic evaluation. Previous studies showed the same results but some of them confirmed that lactate clearance is a better prognostic marker for mortality in critically ill patients compared to initial lactate levels.^15^ Mahmoodpoor et al. showed that lactate and lactate clearance are both useful markers in patients with septic shock. Serum lactate level at 6-hour can be an easier and a more effective marker for septic shock prognosis in patients who were treated with protocol-driven resuscitation bundle therapy.^16^,^17^ On the other hand results of another study showed that Lactate clearance at a discrete time point, seems to be a more reliable prognostic index than the initial lactate value in severe sepsis patients.^18^ Opposite to these results Oh et al. showed that arterial lactate is a very good diagnostic and prognostic predictor of mortality for septic shock. Nevertheless, patients with a high APACHE-II score, high C-reactive protein levels, and chronic heart failure had a poorer prognosis despite a lower arterial lactate level.^19^ Our study showed that initial lactate level was a strong predictor of mortality because our patients had a higher APACHE and initial lactate level. Results of a recently performed multicenter observational study showed that decreasing lactate levels after resuscitation, or lactate clearance is a valuable prognostic marker for mortality in septic patients.^20^ Chertoff et al. showed that lactate clearance during 24 to 48 hours is a prognostic marker for mortality prediction in septic patients.^21^ Chadhaury et al. defined that serial lactate level and a lactate clearance of more than 10% is a good predictor for survival in septic patients.^22^ As fluid overload is an independent risk factor for mortality in critically ill patients, evaluation of fluid responsiveness and volume status is very important is this setting.^23^-^25^ Different studies showed that IVC diameter changes during respiratory cycles, is a predictor of fluid responsiveness. Worapratya et al. showed that Caval index calculated with IVC diameter which is measured by ultrasound in EDs has a good correlation with central venous pressure but not with fluid responsiveness.^26^

Previously, Muller and colleagues defined that, it seems hazardous to manage fluids in a spontaneously breathing patient by using IVC respiratory variations only, until further data are published.^27^ All of the mentioned trials are similar to findings of our study regarding fluid responsiveness and outcome prediction. On the other hand, Preau et al. showed that the collapsibility index of the IVC during a deep inspiration is a simple, noninvasive, bedside technique for prediction of fluid responsiveness in non-intubated patients with sepsis-related acute circulatory failure.^28^ Previously performed studies regarding noninvasive cardiac output monitoring confirmed that minimally-invasive cardiac output monitoring added to usual care, does not facilitate early hemodynamic stabilization in the ICU, nor does it alter the hemodynamic support or outcome. Their role is still controversial and non-invasive monitoring is less intrusive but has not yet been well validated for accuracy compared to gold standard of invasive monitoring.^29^,^30^ As shown, most studies are performed in critically ill ICU patients, but this study is the first one that evaluated the validity of IVC parameter in prediction of mortality in ED.

### Limitation of the study

The most important one is that the lactate clearance was not calculated to be compared to the initial lactate levels. Sample size is the other limitation of our study; as a single center study that was performed on critically ill non-traumatic patients, generalizing the results of this study to routine practice needs future trials. Finally, the method by which we evaluated the cardiac output was not accurate.

## CONCLUSION

Results of our study confirmed that initial lactate value is a useful parameter for prediction of mortality in non-traumatic critically ill patients. IVC diameter changes during spontaneous ventilation and non-invasive cardiac output monitoring do not have acceptable accuracy for prediction of mortality in these patients.

### Authors’ Contributions

Authors contributed as follows to the conception or design of the work; the acquisition, analysis, or interpretation of data for the work; and drafting the work or revising it critically for important intellectual content: **AM and KSh** contributed 30% each, **ASh, MP AT and PM** contributed 10% each. All authors agreed to be accountable for all aspects of the work related to accuracy or integrity of the word.
